# Effects of cognitive ageing trajectories on multiple adverse outcomes among Chinese community-dwelling elderly population

**DOI:** 10.1186/s12877-022-03387-8

**Published:** 2022-08-22

**Authors:** Chao Han, Jing An, Piu Chan

**Affiliations:** 1 National Clinical Research Center for Geriatric Disorders, Xuanwu Hospital of Capital Medical University, Beijing, China; 2 Department of Neurobiology, Neurology and Geriatrics, Beijing Institute of Geriatrics, Clinical Center for Parkinson’s Disease, Key Laboratories for Neurodegenerative Diseases of the Ministry of Education; Beijing Key Laboratory for Parkinson’s Disease, Parkinson Disease Center of Beijing Institute for Brain Disorders, Advanced Innovative Center for Human Brain Protection, Xuanwu Hospital of Capital Medical University, 45 Changchun Road, Beijing, 100053 China

**Keywords:** Cognitive decline, Trajectory, Functional deficits, Chinese, Ageing

## Abstract

**Background:**

Whether cognitive ageing trajectory is related to common functional deficits independent of initial cognitive function remains inconclusive. We aimed to explore the adverse health effect and potential predictive factors of distinct cognitive trajectories among Chinese older adults.

**Methods:**

Three thousand five hundred eighty-one community-dwelling older adults who completed three consecutive cognitive function examinations with the Mini-Mental State Examination (MMSE) over 5 years and were without cognitive impairment at enrollment were included. A group-based trajectory model was used to estimate cognitive ageing trajectories. Multivariable-adjusted odds ratio (OR) and 95% confidence intervals (CI) were computed with logistic regression models to identify potential baseline determinants and health effect of cognitive trajectories on various adverse outcomes.

**Results:**

Two distinct cognitive ageing trajectories were identified with about 5.3% of the study participants ascribed to the rapidly decreasing group. Subjects with rapidly decreasing cognition showed significantly higher odds (OR, 95%CI) of experiencing frailty (4.04, 2.77–5.86), falls (2.01, 1.05–3.70), balance impairment (4.20, 2.75–6.38), high fall risk (5.66, 2.67–11.77) based on the Tinetti total score, disability in activities of daily living (1.76, 1.19–2.56), disability in instrumental activities of daily living (1.52, 1.05–2.19), and motor cognitive risk syndrome (2.24, 1.23–3.98) compared with their steadily decreasing counterparts. Individuals with older age, low education level, no marriage, high score of rapid eye movement behavior disorders, poor physical and cognitive function at baseline were more predisposed to an accelerated cognitive decline.

**Conclusions:**

Faster cognitive decline was independently associated with higher risk of multiple adverse events. Our findings put more emphasis on a routine and constant surveillance of cognitive function among community-dwelling older adults.

**Supplementary Information:**

The online version contains supplementary material available at 10.1186/s12877-022-03387-8.

## Introduction

With rapid population ageing, dementia has become a globally growing public health threat, especially in China which has the largest ageing population and is one of the fastest ageing countries in the world [1–3]. The prevalence and incidence of cognitive impairment ranged from 7.7 to 42.2% and from 21.5 to 80.9 cases per 1000 person-years among Chinese older adults, respectively [4, 5].

Preservation of cognitive function among older adults is a central component of successful ageing [6]. As suggested by numerous studies in recent years, cognitive ageing is a dynamic process with heterogeneous patterns varying across individuals. Longitudinal repeated cognitive assessments might provide opportunities to capture the substantial intra-individual heterogeneity and determine the natural patterns of cognitive ageing over time. This approach would delineate cognitive assessments longitudinally as several cognitive evolving curves. The number of cognitive curves identified in existing studies ranged from two to six while the proportion of individuals assigned to different curves varied widely across these studies [7–11]. However, the trajectory profiles presented in most studies followed a typical pattern of “successful cognitive agers” which started at a high cognitive baseline and exhibited a slow degree of decline, while contrasted this was one or more patterns of “rapid cognitive decliners” that started lower and declined with steeper slopes to varying extents [7–11]. Overall, existing findings have outlined several main phenotypes of cognitive ageing in the general elder population and highlighted the necessity to study cognitive function as trajectories. Besides, it is essential to find out the adverse health effect and predictive characteristics associated with these trajectories among Chinese older adults.

A common methodological limitation of traditional association studies is the use of a single time-point assessment of exposure which overlook the dynamic nature of exposure variations. A newly rising data-driven approach called group-based trajectory modeling (GBTM) has emerged as an informative analytical method that allows grouping subjects presented with similar longitudinal patterns of change [12, 13]. However, even if increasing evidence have demonstrated the detrimental effect of a rapid cognitive decline on several adverse events with this method, including functional disability, hospitalization, nursing home admission, regional brain atrophy, and mortality [14–16], most existing studies failed to take initial cognitive function into account [14, 15]. Furthermore, although cognitive deterioration has been reported to be associated with several functional deficits such as frailty [17], falls [18], rapid eye movement behavior disorders (RBD) [19], depression [20], motor cognitive risk syndrome (MCR) [21] and etc., it remains undetermined whether cognitive ageing trajectory is associated with these functional deficits as well. Likewise, previous efforts which probed into factors shaping cognitive ageing trajectories were limited by small sample size, specific risk factors, or conflicting results primarily from western populations [8, 9, 11, 22–24]. A vast number of factors remain untested among Chinese older adults on this issue. A better understanding of these relationships may help clinicians develop preventive measures to delay and even reverse the development of functional deficits and promote disability-free life expectancy among older adults, namely “healthy ageing” as proposed by the World Health Organization [25].

The Beijing Longitudinal Study on Aging II (BLSA-II) includes a dynamic, prospective, and regionally representative cohort of community-dwelling Chinese elderly population. The main purpose of this study was to investigate whether cognitive ageing trajectories were associated with subsequent common functional deficits within the BLSA-II cohort, including frailty and its components, falls, balance impairment, fall risk, disability in activities of daily living (ADL), disability in instrumental activities of daily living (IADL), RBD, depression, and MCR. In the meanwhile, we also tried to explore potential determinants of these distinct cognitive trajectories in this population.

## Materials and methods

### Study design and participants

The study participants were enrolled from BLSA-II, a large community-based prospective cohort study. Detailed information of this study has been published previously [26]. Briefly, a multistage cluster random sampling method was used to select a representative community-dwelling population aged 55 years or older from three urban and one rural district in Beijing, China. A total of 10,039 participants were recruited at baseline in 2009, among which 7314 and 6399 individuals continued participating the first and second follow-up in 2010–2011 and 2013–2014, respectively. For the present study, we used data from those without cognitive impairment at baseline and fulfilled all three cognitive surveys, leaving 3581 participants in the final analyses. Comparisons between included and excluded participants were shown in Supplemental Table 1. The study protocol was approved by the Research Ethics Committee of Xuanwu Hospital of Capital Medical University. All participants had provided informed consent.

### Measurements

#### Cognitive function

Cognitive function was assessed by trained investigators using the Mini-Mental State Examination (MMSE), which is the most widely used measure of global cognitive function in both clinical and research settings with higher scores indicating better performance (ranges: 0 to 30). Cognitive impairment was defined as: MMSE ≤17 for illiterates; MMSE ≤20 for primary school graduates (≥ 6 years of education); MMSE ≤22 for junior school graduates (≥ 9 years of education); and MMSE ≤23 for college graduates or above (≥ 16 years of education) [27].

#### Frailty

Since the BLSA-II study collected data on frailty status by different means at baseline and the second follow-up, we defined frailty according to frailty index (FI) and frailty phenotype (FP) respectively for these two surveys. For baseline assessment, we selected 34 variables and constructed an index following the standard procedure described by Searle and colleagues (Supplemental Table 2) [28]. FI is the ratio of health deficits present to the total number of deficits considered. Participants scoring 0.25 or above were considered to be frail. For the second follow-up, frailty was defined as a clinical syndrome in which three or more of the following criteria were met according to Fried FP assessment: self-reported unintentional weight loss of 4.5 kg or more in the last year; self-reported exhaustion; low physical activity; weakness (low grip strength); and slow gait [29]. Physical inactivity was defined as no heavy or moderate physical activity and only walk for a few minutes every day. Thresholds of low grip strength was set as < 26 kg for men and < 18 kg for women. Gait speed was calculated through a 15 ft (4.6 m) walking test. Slow gait was defined as gait speed one standard deviation (SD) or more below age and sex-specific mean values of the study population.

#### Falls, balance impairment, and high fall risk

A fall was defined as an unintentional coming to rest on the ground or lower level, with or without loss of consciousness [30]. The subjects were dichotomized into fallers or non-fallers according to a numerical answer greater than or equal to zero to the question “During the preceding 12 months, how many times have you unintentionally lose your balance and land on the ground or lower level?”. Gait and balance performance was assessed with the Tinetti Mobility Test, a valid and reliable tool to measure motor function among elders by expert physicians. The gait subscale grades eight components of gait including gait initiation; step length, height, width, symmetry, continuity; path deviation; and trunk sway. Balance is tested while sitting in a chair, rising up, standing (in both eyes-open and eyes-closed condition), being nudged, turning, and sitting down. The data obtained were represented by three scores: gait score (0–12), balance score (0–16), and total score (0–28) while lower scores indicate worse motor performance and predict a higher risk of falls. Subjects with a Tinetti total score < 24 were generally classified as having balance impairment; those with a total score < 15 were considered at high fall risk.

#### Functional disability

Functional ability was assessed based on the capacity to perform ADL and IADL. ADL covered the ability to perform ten selfcare tasks, including feeding, grooming, dressing, bathing, urine controlling, stool controlling, transferring, ambulating indoors, toilet using, walking up and down stairs. IADL was assessed in a similar fashion based on eight higher-level tasks: cooking, shopping, laundry, doing housework, managing money, taking medicines, riding a bus, and using the telephone. Participants with one or more impaired ADL or IADL were identified as having ADL disability or IADL disability, respectively.

#### RBD

The presence of rapid eye movement behavior disorders (RBD) was determined with a validated RBD questionnaire-Hong Kong (RBDQ-HK). RBDQ-HK was composed of 13 items regarding the presence, frequency, and severity of RBD symptoms, and was assessed on two scales: lifetime occurrence and recent 1-year frequency (Supplemental Table 3). RBDQ-HK demonstrated robust psychometric properties with moderate sensitivity (82.2%) and specificity (86.9%) in screening RBD among general populations [31]. The total RBDQ-HK score was calculated by summing up the scores of all lifetime items (0–20) and recent 1-year frequency items (0–80) with a range from 0 to 100. An RBDQ-HK score of 19 or more can be diagnosed as RBD.

#### Depression

The Geriatric Depression Scale (GDS-30) consisted of 30 items with a dichotomous response of yes or no was used to assess depression in this study (Supplemental Table 4). The score of each item was 1 for the answer indicating depression symptom and 0 for the answer representing non-depression symptom. The total score was obtained by summing up all these items, with a higher score representing greater depressive symptoms. A cutoff value of 11 was used to screen clinical depression as recommended by the developer of GDS-30 and employed in most studies [32, 33].

#### MCR

Motor cognitive risk syndrome (MCR) is a condition characterized by slow gait in company with subjective memory complain in elders without any form of dementia or mobility disability as proposed by Verghese and colleagues [34]. It was diagnosed if participants met all four criteria below: (1) presence of subjective memory complains assessed by using the item “Do you feel that your memory is worse than before?” of the standardized GDS questionnaire; (2) presence of slow gait, as defined above; (3) absence of dementia; (4) preserved ability of four basic ADLs (bathing, dressing, walking, and transferring).

#### Covariates

Standardized structured questionnaires were administered by trained investigators to collect baseline information possibly confounding the main associations of interest in a face-to-face interview. Basic sociodemographic characteristics included age, sex, education level, occupation, marital status and living type (whether living alone). Self-reported smoking and drinking status was uniformly categorized as never or former consumers and current consumers. Physical inactivity was defined as spending no more than half an hour per day on doing moderate-to-rigorous outdoor activities, including brisk walking, running, dancing, hiking, playing ball games, driving a bike and etc. [35]. Sleep duration was measured as self-reported average sleeping hours per night and categorized into < 6 and ≥ 6 hours for analyses [36]. Height and weight were measured with participants wearing light clothes and bare foot. Body mass index (BMI) was calculated by dividing weight in kilograms by height in meters squared, with a threshold of overweight or obesity setting at 24 kg/m^2^ [37]. Family history of dementia and medical histories of common chronic diseases, including stroke, coronary heart disease (CHD), hypertension, diabetes, hyperlipidemia, hyperuricemia, anemia, visual impairment, and hearing impairment were confirmed based on a combination of self-reported physician’s diagnosis, treatment history, and clinical examinations. Nutritional status of participants was also evaluated with mini nutritional assessment (MNA) and divided into non-malnutrition group (MNA ≥ 24) and malnourished or at risk of malnutrition group (MNA < 24) [38].

### Statistical analyses

#### Descriptive analyses

Descriptive statistics were used to summarize the characteristics of participants overall and by subgroups of cognitive trajectories. Continuous variables were described as mean ± standard deviation (SD) and compared by t test, while categorical variables were described as percentages and compared by Chi-square test.

#### Trajectory modelling

To identify distinct cognitive trajectories of the study population, we used GBTM with the SAS macro Proc Traj, which fits a discrete mixture model to identify clusters of longitudinal data series with a maximum-likelihood method [12, 13]. The repeated measurements of MMSE scores over time were modelled as a censored normal distribution using age as a timescale. We tested models with groups ranging from two to five with linear, quadratic, and cubic polynomial function parameters. Selection of the best-fitting trajectory model was based on the Bayesian information criteria (BIC), the average posterior probability of each group membership > 0.7, and a minimal sample size in each trajectory accounted for > 5.0% of total population. As a result, a model fitting two groups with up to cubic order terms of time fits best. These two distinct cognitive trajectories were named according to their visual appearance and clinical meanings: “steadily decreasing” group and “rapidly decreasing” group.

#### Adverse outcomes associated with cognitive trajectories

Multiple logistic regression models were conducted to investigate the associations between GBTM-derived cognitive trajectories and the following adverse outcomes: frailty and its components, falls, balance impairment, fall risk, ADL disability, IADL disability, RBD, depression, and MCR. The minimal model is a univariate model including trajectory groups merely (model 1). Multivariate models further controlled for sociodemographic confounders, health behaviors, comorbidities (model 2), plus baseline MMSE, GDS, and ADL score as appropriate (model 3), and additionally adjusted for baseline levels of targeted outcomes if available (model 4) (Supplemental Table 5). Most adverse outcomes were defined using information obtained at the second follow-up evaluation, except for balance impairment and fall risk which used available results of Tinetti score from the first follow-up instead.

#### Determinants of cognitive trajectories

A multivariate binary logistic regression model was used to distinguish potential baseline determinants of cognitive trajectories by calculating fully adjusted odds ratios (ORs) and corresponding 95% confidence intervals (CIs). This model included all aforementioned potential covariates, and baseline MMSE, GDS, ADL, IADL, RBD, and Tinetti total score as well.

All statistical analyses were performed using the R software (version 3.5.1; R Development Core Team 2018, www.R-project.org) unless specifically noted. Two-tailed *P* values < 0.05 were considered statistically significant.

## Results

**Fig. 1:**
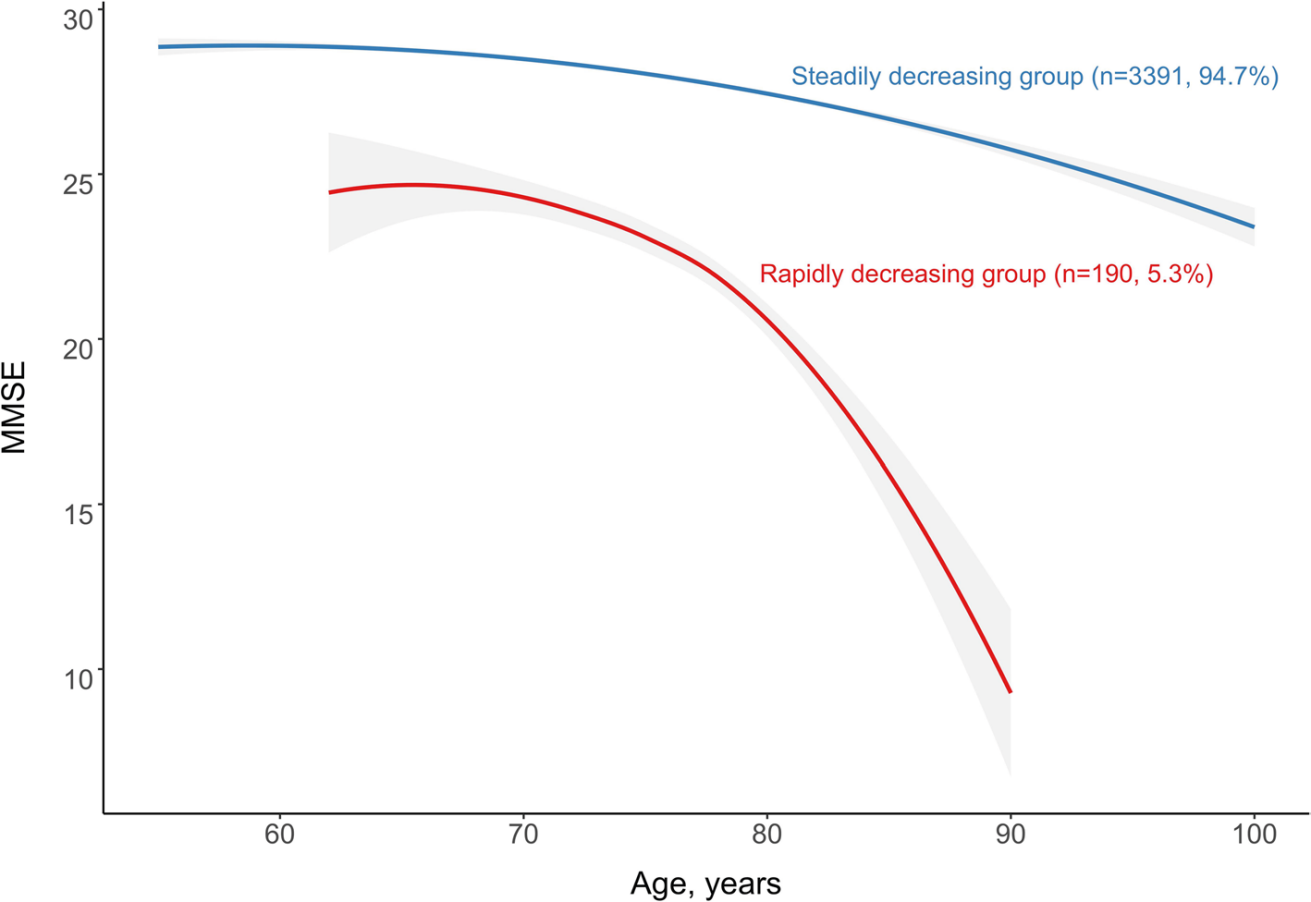
Trajectory modeling identified two cognitive trajectory groups in the BLSA-II cohort of Chinese community-dwelling elderly population. Their patterns by age, the number, and percentage were shown for each group. Grey shades indicate 95% confidence intervals

Trajectory modelling identified two distinct cognitive ageing trajectory groups which could be referred to as “steadily decreasing” group (*n* = 3391, 94.7%) and “rapidly decreasing” group (*n* = 190, 5.3%) (Fig. 1). The steadily decreasing group was characterized by maintaining a relatively high level and a steady decrease of MMSE score with ageing. The rapidly decreasing group was characterized by experiencing an accelerated decrease of MMSE from a moderate initial level. The mean posterior probability of group membership was high for each trajectory group (0.95 and 0.79, respectively).

Baseline characteristics of the study participants by trajectory groups are shown on Table 1. Overall, the average age of included participants was 70.7 (6.5) years old and 60.8% were female. These two cognitive trajectory groups have different sociodemographic and clinical profiles. Compared with the steadily decreasing group, the rapidly decreasing group tended to be older, female, manual workers, never married or non-partnered, sleep-deprived, non-drinkers, and have lower education level. In addition, rates of diabetes, hearing impairment, RBD, ADL or IADL disability, frailty, balance impairment, and being at high fall risk were significantly higher among the rapidly decreasing group (all *P* values < 0.05). However, no significant differences existed in the percentages of smoking, physical inactivity, and other common geriatric diseases across cognitive trajectory groups.

**Table 1:** Baseline characteristics of the study participants stratified by cognitive trajectory group

		Cognitive Trajectory Pattern			
Characteristics	Overall (*n* = 3581)	steadily decreasing (*n* = 3391)	rapidly decreasing (*n* = 190)	Effect Size	*P* Value
Age, y	70.7 ± 6.5	70.5 ± 6.6	73.4 ± 4.5	0.4	< 0.001
Age group				0.1	< 0.001
55–64 years	804 (22.5)	797 (23.5)	7 (3.7)		
65–74 years	1731 (48.3)	1633 (48.2)	98 (51.6)		
75–79 years	739 (20.6)	667 (19.7)	72 (37.9)		
> =80 years	307 (8.6)	294 (8.7)	13 (6.8)		
Female	2179 (60.8)	2032 (59.9)	147 (77.4)	0.1	< 0.001
Education level				0.2	< 0.001
Primary school or lower	1202 (33.6)	1045 (30.8)	157 (82.6)		
Middle or high school	1805 (50.4)	1779 (52.5)	26 (13.7)		
University or higher	574 (16.0)	567 (16.7)	7 (3.7)		
Occupation				0.1	< 0.001
Unemployed	1648 (46.0)	1567 (46.2)	81 (42.6)		
Worker or farmer	1309 (36.6)	1214 (35.8)	95 (50.0)		
Professional technician or others	623 (17.4)	609 (18.0)	14 (7.4)		
Marital status				0.1	< 0.001
Married or partnered	2950 (82.4)	2816 (83.0)	134 (70.5)		
Never married or non-partnered	631 (17.6)	575 (17.0)	56 (29.5)		
Residence type				–	0.311
Living with others	3318 (92.7)	3146 (92.8)	172 (90.5)		
Living alone	263 (7.3)	245 (7.2)	18 (9.5)		
Smoking status				–	0.489
Never or former smoking	3156 (88.2)	2992 (88.3)	164 (86.3)		
Current smoking	424 (11.8)	398 (11.7)	26 (13.7)		
Drinking status				0.04	0.011
Never or former drinking	3181 (88.9)	3001 (88.5)	180 (94.7)		
Current drinking	399 (11.1)	389 (11.5)	10 (5.3)		
Physical activity				–	0.628
< = 30 minutes/day	949 (26.5)	902 (26.6)	47 (24.7)		
> 30 minutes/day	2631 (73.5)	2488 (73.4)	143 (75.3)		
Sleeping habits				0.03	0.049
> = 6 hours	2967 (82.9)	2820 (83.2)	147 (77.4)		
< 6 hours	613 (17.1)	570 (16.8)	43 (22.6)		
Overweight or obese	2258 (63.1)	2129 (62.8)	129 (67.9)	–	0.183
Family history of dementia	65 (1.9)	64 (1.9)	1 (0.5)	–	0.269
Stroke	404 (11.3)	375 (11.1)	29 (15.3)	–	0.096
CHD	596 (16.6)	570 (16.8)	26 (13.7)	–	0.305
Hypertension	1486 (41.5)	1397 (41.2)	89 (46.8)	–	0.144
Diabetes	932 (26.0)	869 (25.6)	63 (33.2)	0.04	0.027
Hyperlipidemia	1566 (43.7)	1473 (43.4)	93 (48.9)	–	0.157
Hyperuricemia	731 (20.4)	684 (20.2)	47 (24.7)	–	0.154
Visual impairment	1594 (44.7)	1496 (44.3)	98 (51.9)	–	0.050
Hearing impairment	1332 (37.3)	1244 (36.8)	88 (46.6)	0.04	0.009
Anemia	83 (2.3)	76 (2.2)	7 (3.7)	–	0.297
Malnourished or at risk of malnutrition	352 (9.8)	327 (9.6)	25 (13.2)	–	0.146
Depression	195 (5.4)	181 (5.3)	14 (7.4)	–	0.300
RBD	107 (3.0)	94 (2.8)	13 (7.0)	0.1	0.002
ADL disability	186 (5.2)	159 (4.7)	27 (14.2)	0.2	< 0.001
IADL disability	404 (11.5)	339 (10.2)	65 (34.9)	0.2	< 0.001
Balance impairment	281 (7.8)	242 (7.1)	39 (20.5)	0.1	< 0.001
At high fall risk	79 (2.2)	68 (2.0)	11 (5.8)	0.1	0.001
Frailty	168 (4.7)	147 (4.3)	21 (11.1)	0.1	< 0.001
MMSE score	27.9 ± 2.5	28.1 ± 2.3	24.3 ± 2.9	1.7	< 0.001
GDS score	2.8 ± 2.4	2.8 ± 2.4	3.2 ± 2.7	0.2	0.008
ADL score	10.2 ± 1.8	10.2 ± 1.6	10.8 ± 4.0	0.4	< 0.001
IADL score	8.7 ± 2.6	8.5 ± 2.2	10.7 ± 5.5	0.9	< 0.001
Frailty index	0.1 ± 0.1	0.1 ± 0.1	0.1 ± 0.1	0.4	< 0.001
Tinetti gait score	11.5 ± 1.6	11.5 ± 1.5	10.8 ± 2.5	0.4	< 0.001
Tinetti balance score	15.2 ± 2.1	15.2 ± 2.0	14.2 ± 3.2	0.5	< 0.001
Tinetti total score	26.7 ± 3.4	26.7 ± 3.2	25.1 ± 5.4	0.5	< 0.001
RBD score	3.6 ± 6.3	3.5 ± 6.2	5.5 ± 7.6	0.3	< 0.001

The effect sizes for significant differences were presented with Cohen’s d for t-test; Φ (Phi) for Chi-square test with a 2 × 2 contingency table; and Cramer’s V for Chi-square test with a table larger than a 2 × 2 contingency table. *Abbreviations: ADL* activities of daily living, *CHD* coronary heart disease, *GDS* Geriatric Depression Scale, *IADL* instrumental activities of daily living, *MCR* motor cognitive risk syndrome, *MMSE* Mini-Mental State Examination, *RBD* rapid eye movement behavior disorders

**Fig. 2:**
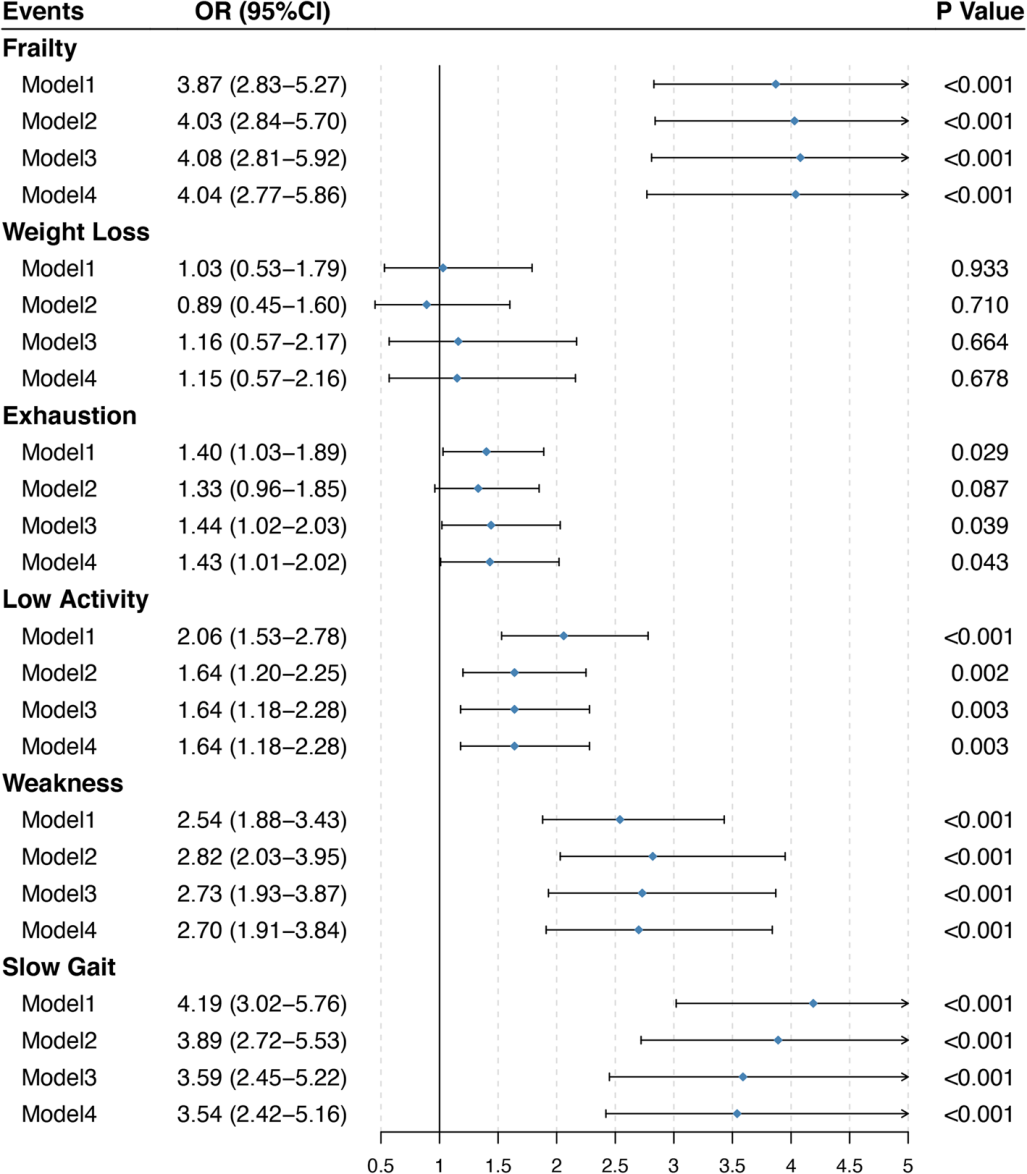
Forest plots of the relative risk of frailty and its component events between the steadily decreasing group and rapidly decreasing group of cognitive function. Model 1: unadjusted model; Model 2: adjusted for all the sociodemographic confounders, health behaviors, and comorbidities; Model 3: adjusted above covariates plus baseline MMSE, GDS, and ADL score; Model 4: adjusted for covariates in model 3 and baseline levels of targeted outcomes. Abbreviations: CI, confidence interval; OR, odds ratio

**Fig. 3:**
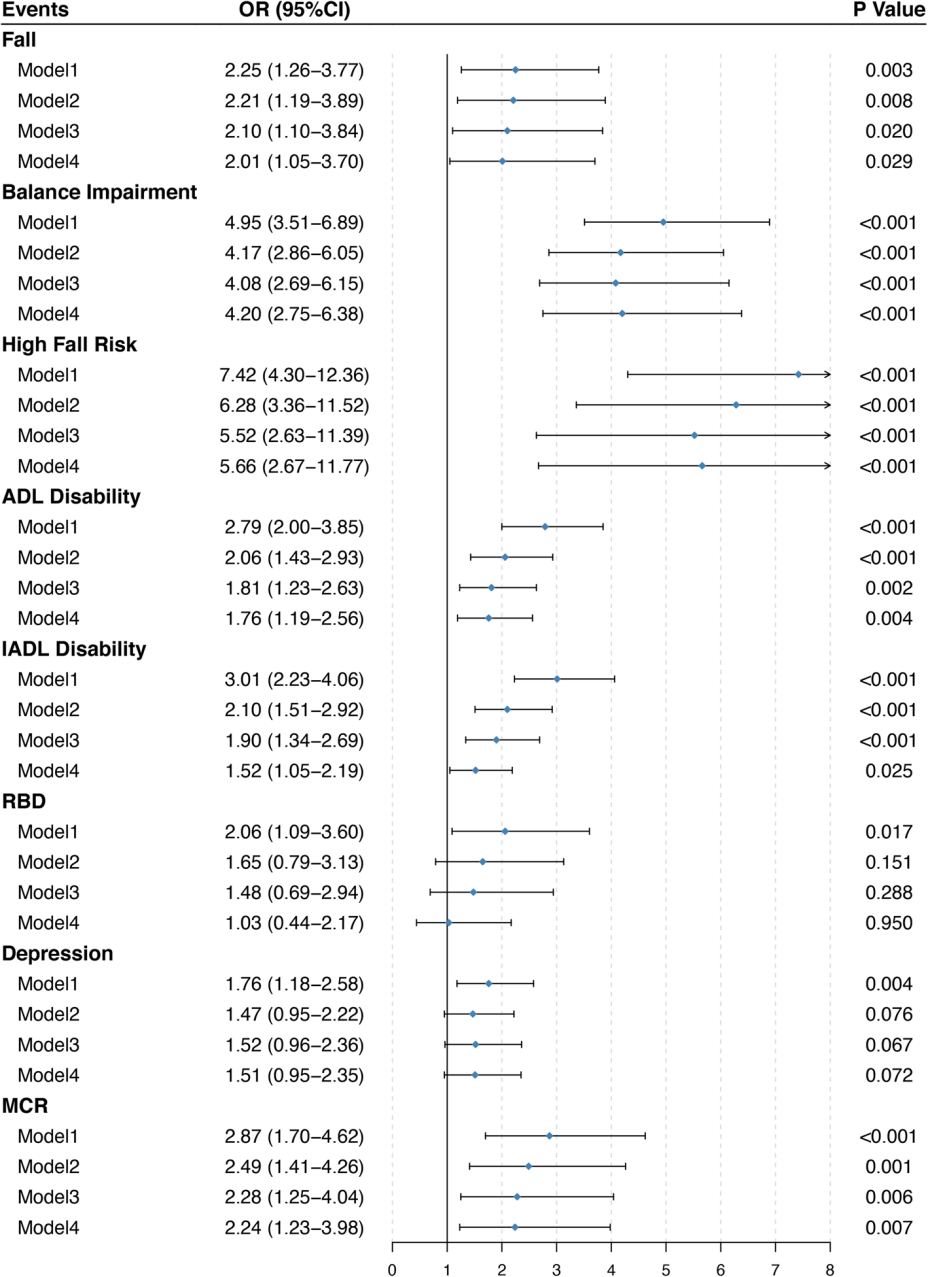
Forest plots of the relative risk of other functional deficits between the steadily decreasing group and rapidly decreasing group of cognitive function. Model 1: unadjusted model; Model 2: adjusted for all the sociodemographic confounders, health behaviors, and comorbidities; Model 3: adjusted for above covariates plus baseline MMSE, GDS, and ADL score as appropriate; Model 4: adjusted for covariates in model 3 and baseline levels of targeted outcomes. Abbreviations: ADL, activities of daily living; CI, confidence interval; IADL, instrumental activities of daily living; MCR, motor cognitive risk syndrome; OR, odds ratio; RBD, rapid eye movement disorders

As expected, subjects with rapidly decreasing cognition showed significantly higher odds of experiencing frailty, falls, balance impairment, high fall risk, ADL disability, IADL disability, and MCR compared with their steadily decreasing counterparts (Figs. 2 and 3). In unadjusted analyses, the ORs (95%CIs) for MCR, frailty and its component events including exhaustion, low activity, weakness, and show gait were 2.87 (1.70, 4.62), 3.87 (2.83, 5.27), 1.40 (1.03, 1.89), 2.06 (1.53, 2.78), 2.54 (1.88, 3.43), and 4.19 (3.02, 5.76), respectively. Further adjustment for subjects’ baseline sociodemographic characteristics, health behaviors, comorbidities, MMSE score, GDS score, ADL score, and baseline levels of targeted outcomes slightly modified the ORs as 2.24 (1.23, 3.98), 4.04 (2.77, 5.86), 1.43 (1.01, 2.02), 1.64 (1.18, 2.28), 2.70 (1.91, 3.84), 3.54 (2.42, 5.16), respectively. Similarly, the unadjusted ORs for other adverse outcomes were attenuated but remained significant after adjusting for confounding factors. In brief, an accelerated cognitive decline was associated with significantly higher odds of developing falls (2.01, 1.05–3.70), balance impairment (4.20, 2.75–6.38), high fall risk (5.66, 2.67–11.77), ADL disability (1.76, 1.19–2.56), and IADL disability (1.52, 1.05–2.19) independent of initial cognitive function, respectively. Whereas we failed to find any significant risk differences between two cognitive trajectories in terms of weight loss, RBD and depression.

In the analyses to detect independent determinants of cognitive decline pattern, only ageing (OR, 95%CI: 2.35, 1.08–5.92 for 65–74 years old group), education level (OR, 95%CI: 0.23, 0.14–0.38 for middle or high school group; 0.28, 0.11–0.64 for university or higher group), marital status (OR, 95%CI: 1.58, 1.01–2.44), baseline MMSE score (OR, 95%CI: 0.66, 0.61–0.71), IADL score (OR, 95%CI: 1.18, 1.12–1.24), and RBD score (OR, 95%CI: 1.04, 1.01–1.06) remained significant after adjusting for other confounding factors (Table 2).

**Table 2:** Multivariate-adjusted ORs and 95%CI of baseline characteristics for rapidly decreasing group compared with steadily decreasing group of cognitive function

Characteristics	OR (95%CI)	*P* Value
Age group		
55–64 years	reference	
65–74 years	2.35 (1.08–5.92)	0.046
75–79 years	1.74 (0.76–4.54)	0.219
> =80 years	0.32 (0.10–1.04)	0.053
Female	0.86 (0.54–1.41)	0.545
Education level		
Primary school or lower	reference	
Middle or high school	0.23 (0.14–0.38)	< 0.001
University or higher	0.28 (0.11–0.64)	0.005
Occupation		
Unemployed	reference	
Worker or farmer	0.76 (0.51–1.15)	0.200
Professional technician or others	0.64 (0.30–1.27)	0.221
Marital status		
Married or partnered	reference	
Never married or non-partnered	1.58 (1.01–2.44)	0.042
Residence type		
Living with others	reference	
Living alone	0.68 (0.34–1.28)	0.246
Smoking status		
Never or former smoking	reference	
Current smoking	1.43 (0.78–2.54)	0.234
Drinking status		
Never or former smoking	reference	
Current drinking	0.56 (0.23–1.21)	0.163
Physical activity		
<= 30 minutes/day	reference	
> 30 minutes/day	1.10 (0.72–1.70)	0.669
Sleeping habits		
> = 6 hours	reference	
< 6 hours	0.88 (0.56–1.34)	0.552
Overweight or obese	1.04 (0.71–1.55)	0.840
Family history of dementia	0.39 (0.02–2.02)	0.371
Stroke	1.20 (0.69–2.02)	0.506
CHD	0.90 (0.52–1.51)	0.699
Hypertension	1.22 (0.83–1.78)	0.316
Diabetes	1.02 (0.68–1.52)	0.905
Hyperlipidemia	1.27 (0.89–1.83)	0.190
Hyperuricemia	0.97 (0.63–1.47)	0.898
Visual impairment	0.86 (0.54–1.35)	0.503
Hearing impairment	0.88 (0.56–1.37)	0.563
Anemia	0.89 (0.26–2.52)	0.833
Malnourished or at risk of malnutrition	0.97 (0.54–1.67)	0.905
Frailty	1.59 (0.67–3.62)	0.281
MMSE score	0.66 (0.61–0.71)	< 0.001
GDS score	1.02 (0.94–1.10)	0.639
ADL score	0.99 (0.91–1.06)	0.704
IADL score	1.18 (1.12–1.24)	< 0.001
Tinetti total score	1.01 (0.97–1.06)	0.650
RBD score	1.04 (1.01–1.06)	0.002

The full model adjusted for age, sex, education level, occupation, marital status, residence type, smoking status, drinking status, physical activity, sleeping habits, BMI, family history of dementia, various clinical comorbidities (stroke, CHD, hypertension, diabetes, hyperlipidemia, hyperuricemia, visual impairment, hearing impairment, anemia), nutritional status, frailty status, MMSE, GDS, ADL, IADL, RBD, and Tinetti total score

*Abbreviations: ADL* activities of daily living, *CHD* coronary heart disease, *CI* confidence interval, *GDS* Geriatric Depression Scale, *IADL* instrumental activities of daily living, *MCR* motor cognitive risk syndrome, *MMSE* Mini-Mental State Examination, *OR* odds ratio, *RBD* rapid eye movement behavior disorders

## Discussion

We observed two heterogeneous cognitive trajectories in a large prospective community-based cohort of 3581 old adults during a 5-year follow-up. A rapid cognitive decline was associated with a significantly higher risk of multiple adverse outcomes including frailty, falls, balance impairment, high fall risk, ADL disability, IADL disability, and MCR independent of initial cognitive function. In addition, we proved that individuals with older age, low education level, no marriage, high RBD score, poor physical and cognitive function at baseline were more predisposed to an accelerated cognitive decline in the following years.

In line with previous studies which consistently identified two to six cognitive trajectories, the steady decline trajectory accounted for most of the study participants, while a precipitous decline only accounted for 2.8 to 18.9% [7–11]. This indicates that the majority of general older adults are cognitively stable through their ageing process while an accelerating decline is relatively uncommon. In addition, prior studies have provided some evidence for the detrimental effect of cognitive decline on various adverse events in elderly, such as dementia, frailty, disability, mortality, and etc. [39–41]. However, most of them were based on a single and conventional measurement of cognition while data on long-term cognitive changes are scarce. Our study focused on dynamic cognitive changes over time and provided more insight into evolving risk. We have proved that long-term cognitive changing trajectory is an independent predictor of various adverse events beyond initial cognitive levels and may help to identify elders at high risk of frailty, falls, and functional disability that require intervention in the future. Recently, emerging studies have linked the cognitive trajectory to hospitalization, nursing home admission, regional brain atrophy, and mortality among older adults of different ethnicities as well [14–16, 42].

Frailty and cognitive impairment are ranked as the two most common geriatric disorders. The mutual effect of cognitive decline and physical frailty have been described by other researchers as mentioned above [39, 43]. Our study adds to literature by providing a more nuanced understanding of the association between cognitive ageing trajectory with physical frailty and its components, as well as MCR, a recently proposed pre-dementia syndrome characterized by subjective memory complaints and slow gait. Physical function, such as ADL and IADL, is a good measurement of functional capacity and a proxy of health status widely used in ageing studies. Our results prove that low physical function might increase the odds of accelerated cognitive decline and vice versa, which implies that the association of physical function and cognitive decline is likely to be bidirectional and interactive as well. Although potential mechanisms of these inter-relationships have not been fully elucidated, it is plausible that cognitive decline and physical dysfunction share common underlying pathologies. Several pathophysiological factors might be involved, including Alzheimer’s disease-related plaque development, oxidative stress, chronic inflammation, imbalanced energy metabolism, micronutrient deficiencies, epigenic changes, and cardiovascular diseases [39, 44, 45]. With regards to falls, our results further validate the enormous impact of cognitive decline not only on falls events but also on balance impairment and high fall risk measured with Tinetti scores. Increasing studies support the interrelationship of slower gait or gait instability with cognitive deterioration [46–48]. The simultaneous impairment of cognitive function and balance regulation may be partially explained by the damage in shared brain regions or networks essential for planning and monitoring goal-directed behaviors [49, 50].

Besides, our study has revealed several key determinants of heterogeneous cognitive trajectories. Among them, advanced age, low education level and cognitive reserve, as the most commonly identified risk factors of dementia, have been shown to be important predictors of faster cognitive decline [8, 22, 43, 51]. In addition to baseline cognitive function, a notable difference in physical function was also observed across trajectory groups: older adults with IADL deficits tended to have a higher risk of being on the rapid decline trajectory. This is in agreement with the results derived from an European cohort study conducted at 78 cancer sites [52] and a Taiwan longitudinal study on aging [23]. In addition, our results indicated that the relationship between sleep disorders like RBD and cognitive deterioration was not exclusive to patients with Parkinson’s disease but possibly existed among general older adults [53]. According to recent studies, elders’ cognitive progressive pattern was also shaped by gender, sleep duration, social engagement, diabetes, depression, genetic factors and etc. [7, 8, 43, 54], some of which haven’t been replicated in this study. Inconsistencies might be caused by different measurements of cognitive performance, heterogeneous characteristic of study participants, varying adjusting factors, disparity in sample size and study design. Overall, these findings align with current knowledge about risk and protective factors for cognitive ageing [1]. From a public health perspective, our results further highlight the importance of education attainment in the preservation of cognitive function among older adults.

To our knowledge, this is the first study to systematically explore the association between cognitive changing patterns and various functional deficits among healthy older adults using trajectory analyses. Additionally, the use of longitudinal data from a well-established cohort enabled us to probe into the true causal associations after adjusting for substantial widely recognized confounding factors. Our study has several potential clinical implications. Firstly, this could be deemed as a meaningful attempt to investigate easily accessed predictors of cognitive ageing patterns. It indicated that subjects with specific baseline characteristics like advanced age, no marriage, low education level, sleep disorder, poor physical and cognitive function are prone to a rapid cognitive decline. This will aid clinicians in counseling patients or caregivers on possible cognitive prognosis and establishing more tailored treatment strategies. Moreover, our findings of a strong temporal association between cognitive changing pattern with various health outcomes have important implications for medical policy and practice aimed at promoting “healthy ageing”. As longevity is increasing, policy makers should launch more government programs on slowing down cognitive decline among the aging population to prevent, delay, and even reverse the development of subsequent functional deficits. Meanwhile, there is a call for building a routine and constant surveillance network of cognitive function among community-dwelling elderly population since a single-time assessment is insufficient to detect individuals at high risk [55].

Nevertheless, these results should also be interpreted in light of some limitations. Firstly, we only included Chinese community-dwelling older adults in this study, which may limit the generalizability of these trajectories and association results to other ethnic populations. Whereas the homogeneous nature of our cohort may result in few confounding bias and enhanced internal validity. Secondly, since a less functionally and cognitively impaired sample was enrolled in our analyses, we might underestimate the proportion of participants who showed a markedly accelerated cognitive decline. Thirdly, even though we have collected comprehensive baseline information through standardized protocols and stringent quality control procedures, the possibility of residual confounding by some unavailable risk factors can’t be ruled out. Furthermore, we only collected three waves of cognitive assessment throughout 5 years. Data gathered over longer observation periods with more frequent assessments may generate more accurate results.

## Conclusions

In conclusion, our study showed that Chinese community-living older adults followed two distinct cognitive ageing trajectories. A rapid cognitive decline was independently associated with substantially higher risk of multiple adverse outcomes. Individuals with older age, low education level, no marriage, high RBD score, poor physical and cognitive function at baseline were more predisposed to an accelerated cognitive decline in the following years. A close and serial monitoring of cognitive performance among older adults appears to be a practical and economical tool to promote healthy ageing in the long term. More large-scale and well-designed longitudinal studies are warranted to validate our findings in the future.

## Supplementary Information

**Additional file 1.**

**Additional file 2.**

**Additional file 3.**

**Additional file 4.**

**Additional file 5.**

## Data Availability

The datasets analyzed during the current study are not publicly available due to privacy/ethical restrictions but are available from the corresponding author on reasonable request.

## Abbreviations

MMSE: Mini-Mental State Examination
OR: odds ratio
CI: confidence interval
GBTM: group-based trajectory modeling
BLSA-II: Beijing Longitudinal Study on Aging II
ADL: disability in activities of daily living
IADL: disability in instrumental activities of daily living
RBD: rapid eye movement behavior disorders
MCR: motor cognitive risk syndrome
FI: frailty index
FP: frailty phenotype
SD: standard deviation
RBDQ-HK: RBD questionnaire-Hong Kong
GDS: Geriatric Depression Scale
BMI: body mass index
CHD: coronary heart disease
MNA: mini nutritional assessment
BIC: Bayesian information criteria
