# Supplementary material for: Effects of cognitive ageing trajectories on multiple adverse outcomes among Chinese community-dwelling elderly population
Source: BMC Geriatr. 2022 Aug 22;22:692. doi: 10.1186/s12877-022-03387-8 (PMC9396872; doi:10.1186/s12877-022-03387-8)
Supplement: Supplementary file 1 — Additional file 1. [file 12877_2022_3387_MOESM1_ESM.docx]

**Supplemental Table1. Baseline characteristics of included versus excluded participants.**

| Characteristics | Overall  (n=10375) | Included  (n=3581) | Excluded  (n=6794) | P Value |
| --- | --- | --- | --- | --- |
| Age, y | 70.1 ± 7.7 | 70.7 ± 6.5 | 69.7 ± 8.3 | <0.001 |
| Age group |  |  |  | <0.001 |
| 55-64 years | 2729 (27.2) | 804 (22.5) | 1925 (29.8) |  |
| 65-74 years | 4306 (42.9) | 1731 (48.3) | 2575 (39.9) |  |
| 75-79 years | 1938 (19.3) | 739 (20.6) | 1199 (18.6) |  |
| >=80 years | 1066 (10.6) | 307 (8.6) | 759 (11.8) |  |
| Female | 6153 (61.3) | 2179 (60.8) | 3974 (61.5) | 0.512 |
| Education level |  |  |  | <0.001 |
| Primary school or lower | 3822 (38.7) | 1202 (33.6) | 2620 (41.6) |  |
| Middle or high school | 4781 (48.4) | 1805 (50.4) | 2976 (47.2) |  |
| University or higher | 1281 (13.0) | 574 (16.0) | 707 (11.2) |  |
| Occupation |  |  |  | <0.001 |
| Unemployed | 3053 (30.9) | 1648 (46.0) | 1405 (22.3) |  |
| Worker or farmer | 5115 (51.7) | 1309 (36.6) | 3806 (60.4) |  |
| Professional technician or others | 1718 (17.4) | 623 (17.4) | 1095 (17.4) |  |
| Marital status |  |  |  | 0.018 |
| Married or partnered | 8079 (81.1) | 2950 (82.4) | 5129 (80.4) |  |
| Never married or non-partnered | 1880 (18.9) | 631 (17.6) | 1249 (19.6) |  |
| Residence type |  |  |  | 0.161 |
| Living with others | 9176 (92.1) | 3318 (92.7) | 5858 (91.8) |  |
| Living alone | 783 (7.9) | 263 (7.3) | 520 (8.2) |  |
| Smoking status |  |  |  | 0.013 |
| Never or former smoking | 8610 (87.0) | 3156 (88.2) | 5454 (86.4) |  |
| Current smoking | 1283 (13.0) | 424 (11.8) | 859 (13.6) |  |
| Drinking status |  |  |  | <0.001 |
| Never or former smoking | 8586 (86.8) | 3181 (88.9) | 5405 (85.6) |  |
| Current drinking | 1307 (13.2) | 399 (11.1) | 908 (14.4) |  |
| Physical activity |  |  |  | <0.001 |
| <= 30 minutes/day | 2249 (22.7) | 949 (26.5) | 1300 (20.6) |  |
| > 30 minutes/day | 7644 (77.3) | 2631 (73.5) | 5013 (79.4) |  |
| Sleeping habits |  |  |  | 0.651 |
| >= 6 hours | 8175 (82.6) | 2967 (82.9) | 5208 (82.5) |  |
| < 6 hours | 1718 (17.4) | 613 (17.1) | 1105 (17.5) |  |
| Overweight or obese | 6119 (61.0) | 2258 (63.1) | 3861 (59.8) | 0.001 |
| Family history of dementia | 155 (1.6) | 65 (1.9) | 90 (1.4) | 0.144 |
| Stroke | 1058 (10.5) | 404 (11.3) | 654 (10.1) | 0.077 |
| CHD | 1314 (13.1) | 596 (16.6) | 718 (11.1) | <0.001 |
| Hypertension | 4255 (42.4) | 1486 (41.5) | 2769 (42.9) | 0.187 |
| Diabetes | 2519 (25.1) | 932 (26.0) | 1587 (24.6) | 0.113 |
| Hyperlipidemia | 4288 (42.7) | 1566 (43.7) | 2722 (42.1) | 0.130 |
| Hyperuricemia | 1844 (18.4) | 731 (20.4) | 1113 (17.2) | <0.001 |
| Visual impairment | 4148 (41.7) | 1594 (44.7) | 2554 (40.1) | <0.001 |
| Hearing impairment | 3273 (32.9) | 1332 (37.3) | 1941 (30.5) | <0.001 |
| Anemia | 248 (2.5) | 83 (2.3) | 165 (2.6) | 0.393 |
| Malnourished or at risk of malnutrition | 1124 (11.2) | 352 (9.8) | 772 (12.0) | 0.001 |
| Depression | 471 (4.7) | 195 (5.4) | 276 (4.3) | 0.009 |
| RBD | 224 (3.2) | 107 (3.0) | 117 (3.3) | 0.535 |
| ADL disability | 685 (6.8) | 186 (5.2) | 499 (7.7) | <0.001 |
| IADL disability | 999 (14.2) | 404 (11.5) | 595 (17.0) | <0.001 |
| Balance impairment | 895 (8.9) | 281 (7.8) | 614 (9.5) | 0.006 |
| At high fall risk | 337 (3.4) | 79 (2.2) | 258 (4.0) | <0.001 |
| Frailty | 540 (5.4) | 168 (4.7) | 372 (5.8) | 0.026 |
| MMSE score | 26.8 ± 4.0 | 27.9 ± 2.5 | 26.2 ± 4.5 | <0.001 |
| GDS score | 2.6 ± 2.3 | 2.8 ± 2.4 | 2.5 ± 2.3 | <0.001 |
| ADL score | 10.4 ± 2.4 | 10.2 ± 1.8 | 10.4 ± 2.6 | <0.001 |
| IADL score | 8.9 ± 3.2 | 8.7 ± 2.6 | 9.2 ± 3.7 | <0.001 |
| Frailty index | 0.1 ± 0.1 | 0.1 ± 0.1 | 0.1 ± 0.1 | 0.304 |
| Tinetti gait score | 11.4 ± 1.9 | 11.5 ± 1.6 | 11.3 ± 2.1 | <0.001 |
| Tinetti balance score | 15.1 ± 2.4 | 15.2 ± 2.1 | 15.1 ± 2.6 | 0.183 |
| Tinetti total score | 26.5 ± 4.2 | 26.7 ± 3.4 | 26.4 ± 4.5 | 0.001 |
| RBD score | 4.0 ± 6.7 | 3.6 ± 6.3 | 4.4 ± 7.0 | <0.001 |

Abbreviations: ADL, activities of daily living; CHD, coronary heart disease; GDS, Geriatric Depression Scale; IADL, instrumental activities of daily living; MCR, motor cognitive risk syndrome; MMSE, Mini-Mental State Examination; RBD, rapid eye movement behavior disorders.
