# Supplementary material for: Effects of cognitive ageing trajectories on multiple adverse outcomes among Chinese community-dwelling elderly population
Source: BMC Geriatr. 2022 Aug 22;22:692. doi: 10.1186/s12877-022-03387-8 (PMC9396872; doi:10.1186/s12877-022-03387-8)
Supplement: Supplementary file 2 — Additional file 2. [file 12877_2022_3387_MOESM2_ESM.docx]

**Supplemental Table2. List of variables used to construct the frailty index.**

|  | Variables | Coding |
| --- | --- | --- |
| 1 | Self-reported health | Good=0, general=0.5, bad=1 |
| 2 | Hypertension | No=0, Yes=1 |
| 3 | Coronary heart disease | No=0, Yes=1 |
| 4 | Stroke | No=0, Yes=1 |
| 5 | Other cerebrovascular disease | No=0, Yes=1 |
| 6 | Diabetes | No=0, Yes=1 |
| 7 | Hyperlipidemia | No=0, Yes=1 |
| 8 | Hyperuricemia | No=0, Yes=1 |
| 9 | Overweight and obesity | No=0, Yes=1 |
| 10 | Osteoarthritis | No=0, Yes=1 |
| 11 | Cancer | No=0, Yes=1 |
| 12 | Cataract | No=0, Yes=1 |
| 13 | Deaf | No=0, Yes=1 |
| 14 | Chronic bronchitis | No=0, Yes=1 |
| 15 | Kidney disease | No=0, Yes=1 |
| 16 | Scapulohumeral periarthritis | No=0, Yes=1 |
| 17 | Chronic lumbocrural pain | No=0, Yes=1 |
| 18 | Anemia | No=0, Yes=1 |
| 19 | Malnutrition | No=0, Yes=1 |
| 20 | Limb disability | No=0, Yes=1 |
| 21 | Depression | No=0, Yes=1 |
| 22 | Life is interesting | Yes=0, General=0.5, No=1 |
| 23 | Health satisfaction | Yes=0, General=0.5, No=1 |
| 24 | Life status satisfaction | Yes=0, General=0.5, No=1 |
| 25 | Feeding | Independent=0, Difficulty=0.5, Dependent=1 |
| 26 | Bathing | Independent=0, Difficulty=0.5, Dependent=1 |
| 27 | Grooming | Independent=0, Difficulty=0.5, Dependent=1 |
| 28 | Dressing | Independent=0, Difficulty=0.5, Dependent=1 |
| 29 | Controlling bowels | Independent=0, Difficulty=0.5, Dependent=1 |
| 30 | Controlling bladder | Independent=0, Difficulty=0.5, Dependent=1 |
| 31 | Toileting | Independent=0, Difficulty=0.5, Dependent=1 |
| 32 | Transferring bed | Independent=0, Difficulty=0.5, Dependent=1 |
| 33 | Walk 45 meters | Independent=0, Difficulty=0.5, Dependent=1 |
| 34 | Climbing stairs | Independent=0, Difficulty=0.5, Dependent=1 |
