# Supplementary material for: Effects of cognitive ageing trajectories on multiple adverse outcomes among Chinese community-dwelling elderly population
Source: BMC Geriatr. 2022 Aug 22;22:692. doi: 10.1186/s12877-022-03387-8 (PMC9396872; doi:10.1186/s12877-022-03387-8)
Supplement: Supplementary file 3 — Additional file 3. [file 12877_2022_3387_MOESM3_ESM.docx]

**Supplemental Table3. The detailed questions of RBD questionnaire-Hong Kong (RBDQ-HK) in English version.**

| **Symptoms** | **Lifetime occurrence**  (Did you have the following RBD symptom?) | | | **Recent one-year frequency**  **(**Did it happen in the recent one-year?**)** | | | | |
| --- | --- | --- | --- | --- | --- | --- | --- | --- |
| 1. Did you often have dreams? | □ I don’t remember/  I don’t know | □ No | □ Yes | □ No | □ Once or few times per year | □ Once or few times per month | □ One to two times per week | □ Three times or above per weeks |
| 2. Did you often have nightmares? | □ I don’t remember/  I don’t know | □ No | □ Yes | □ No | □ Once or few times per year | □ Once or few times per month | □ One to two times per week | □ Three times or above per weeks |
| 3. Did you have dreams with an emotional and sorrowful content? | □ I don’t remember/  I don’t know | □ No | □ Yes | □ No | □ Once or few times per year | □ Once or few times per month | □ One to two times per week | □ Three times or above per weeks |
| 4. Did you have dreams with a violent or aggressive content (e.g., fighting with someone)? | □ I don’t remember/  I don’t know | □ No | □ Yes | □ No | □ Once or few times per year | □ Once or few times per month | □ One to two times per week | □ Three times or above per weeks |
| 5. Did you have dreams with a frightening and horrifying content (e.g., being chased by ghost)? | □ I don’t remember/  I don’t know | □ No | □ Yes | □ No | □ Once or few times per year | □ Once or few times per month | □ One to two times per week | □ Three times or above per weeks |
| 6. Did you have sleep talking? | □ I don’t remember/  I don’t know | □ No | □ Yes | □ No | □ Once or few times per year | □ Once or few times per month | □ One to two times per week | □ Three times or above per weeks |
| 7. Did you shout, yell or swear during your sleep? | □ I don’t remember/  I don’t know | □ No | □ Yes | □ No | □ Once or few times per year | □ Once or few times per month | □ One to two times per week | □ Three times or above per weeks |
| 8. Did you move your arms or legs in response to your dream contents during sleep? | □ I don’t remember/  I don’t know | □ No | □ Yes | □ No | □ Once or few times per year | □ Once or few times per month | □ One to two times per week | □ Three times or above per weeks |
| 9. Have you ever fallen from your bed? | □ I don’t remember/  I don’t know | □ No | □ Yes | □ No | □ Once or few times per year | □ Once or few times per month | □ One to two times per week | □ Three times or above per weeks |
| 10. Have you ever hurt yourself or your bed-partner while you were sleeping? | □ I don’t remember/  I don’t know | □ No | □ Yes | □ No | □ Once or few times per year | □ Once or few times per month | □ One to two times per week | □ Three times or above per weeks |
| 11. Have you ever *attempted to* assault your bed-partner or *almost* hurt yourself while you were sleeping? | □ I don’t remember/  I don’t know | □ No | □ Yes | □ No | □ Once or few times per year | □ Once or few times per month | □ One to two times per week | □ Three times or above per weeks |
| 12. Did the scenario described in 10 or 11 relate to your dream contents? | □ I don’t remember/  I don’t know | □ No | □ Yes | □ No | □ Once or few times per year | □ Once or few times per month | □ One to two times per week | □ Three times or above per weeks |
| 13. Did the situations described above disturb your sleep? | □ I don’t remember/  I don’t know | □ No | □ Yes | □ No | □ Once or few times per year | □ Once or few times per month | □ One to two times per week | □ Three times or above per weeks |

Instructions: For each lifetime item, Q1-Q5 and Q13 were scored as follows: “don’t know” = 0, “no” = 0, “yes” = 1; Q6-Q12 were additionally weighted: “don’t know” = 0, “no” = 0, “yes” = 2. For each recent one-year frequency item with a five-point scale, Q1-Q5 and Q13 were scored as follows: “no” = 0, “yes/once or few times per year” = 1, “once or few times per month” = 2, “1–2 times per week” = 3, “3 times or above per week” = 4; Q6-Q12 were additionally weighted: “no” = 0, “yes/once or few times per year” = 2, “once or few times per month” = 4, “1–2 times per week” = 6, “3 times or above per week” = 8.
