# Supplementary material for: Effects of cognitive ageing trajectories on multiple adverse outcomes among Chinese community-dwelling elderly population
Source: BMC Geriatr. 2022 Aug 22;22:692. doi: 10.1186/s12877-022-03387-8 (PMC9396872; doi:10.1186/s12877-022-03387-8)
Supplement: Supplementary file 4 — Additional file 4. [file 12877_2022_3387_MOESM4_ESM.docx]

**Supplemental Table4. The Questions of Geriatric Depression Scale (GDS-30).**

Instructions: choose the best answer for how you felt over the past week.

| No. | Question | Answer | Score |
| --- | --- | --- | --- |
| 1 | Are you basically satisfied with your life? | yes/no |  |
| 2 | Have you dropped many of your activities and interests? | yes/no |  |
| 3 | Do you feel that your life is empty? | yes/no |  |
| 4 | Do you often get bored? | yes/no |  |
| 5 | Are you hopeful about the future? | yes/no |  |
| 6 | Are you bothered by thoughts you can’t get out of your head? | yes/no |  |
| 7 | Are you in good spirits most of the time? | yes/no |  |
| 8 | Are you afraid that something bad is going to happen to you? | yes/no |  |
| 9 | Do you feel happy most of the time? | yes/no |  |
| 10 | Do you often feel helpless? | yes/no |  |
| 11 | Do you often get restless and fidgety? | yes/no |  |
| 12 | Do you prefer to stay at home, rather than going out and doing new things? | yes/no |  |
| 13 | Do you frequently worry about the future? | yes/no |  |
| 14 | Do you feel you have more problems with memory than most? | yes/no |  |
| 15 | Do you think it is wonderful to be alive now? | yes/no |  |
| 16 | Do you often feel downhearted and blue? | yes/no |  |
| 17 | Do you feel pretty worthless the way you are now? | yes/no |  |
| 18 | Do you worry a lot about the past? | yes/no |  |
| 19 | Do you find life very exciting? | yes/no |  |
| 20 | Is it hard for you to get started on new projects? | yes/no |  |
| 21 | Do you feel full of energy? | yes/no |  |
| 22 | Do you feel that your situation is hopeless? | yes/no |  |
| 23 | Do you think that most people are better off than you are? | yes/no |  |
| 24 | Do you frequently get upset over little things? | yes/no |  |
| 25 | Do you frequently feel like crying? | yes/no |  |
| 26 | Do you have trouble concentrating? | yes/no |  |
| 27 | Do you enjoy getting up in the morning? | yes/no |  |
| 28 | Do you prefer to avoid social gatherings? | yes/no |  |
| 29 | Is it easy for you to make decisions? | yes/no |  |
| 30 | Is your mind as clear as it used to be? | yes/no |  |
| Total score | | |  |

Note: Q1, Q5, Q7, Q9, Q15, Q19, Q21, Q27, Q29 and Q30 were scored as follows: “no” = 1, “yes” = 0; Q2-Q4, Q6, Q8, Q10-Q14, Q16-Q18, Q20, Q22-Q26 and Q28 were scored as follows: “no” = 0, “yes” = 1.
