# Supplementary material for: Effects of cognitive ageing trajectories on multiple adverse outcomes among Chinese community-dwelling elderly population
Source: BMC Geriatr. 2022 Aug 22;22:692. doi: 10.1186/s12877-022-03387-8 (PMC9396872; doi:10.1186/s12877-022-03387-8)
Supplement: Supplementary file 5 — Additional file 5. [file 12877_2022_3387_MOESM5_ESM.docx]

**Supplemental Table5. Variables included in the logistic regression models for each adverse outcome.**

| Outcomes | Model1 | Model2 | Model3 | Model4 |
| --- | --- | --- | --- | --- |
| Frailty and its components | trajectory groups | trajectory groups, sociodemographic confounders, health behaviors, comorbidities | trajectory groups,  sociodemographic confounders, health behaviors, comorbidities,  baseline MMSE, GDS, and ADL score | trajectory groups,  sociodemographic confounders, health behaviors, comorbidities,  baseline MMSE, GDS, and ADL score, plus baseline frailty status |
| Falls | trajectory groups | trajectory groups, sociodemographic confounders, health behaviors, comorbidities | trajectory groups,  sociodemographic confounders, health behaviors, comorbidities,  baseline MMSE, GDS, and ADL score | trajectory groups,  sociodemographic confounders, health behaviors, comorbidities,  baseline MMSE, GDS, and ADL score, plus baseline fall status |
| Balance impairment | trajectory groups | trajectory groups, sociodemographic confounders, health behaviors, comorbidities | trajectory groups,  sociodemographic confounders, health behaviors, comorbidities,  baseline MMSE, GDS, and ADL score | trajectory groups,  sociodemographic confounders, health behaviors, comorbidities,  baseline MMSE, GDS, and ADL score, plus baseline Tinetti total score |
| Fall risk | trajectory groups | trajectory groups, sociodemographic confounders, health behaviors, comorbidities | trajectory groups,  sociodemographic confounders, health behaviors, comorbidities,  baseline MMSE, GDS, and ADL score | trajectory groups,  sociodemographic confounders, health behaviors, comorbidities,  baseline MMSE, GDS, and ADL score, plus baseline Tinetti total score |
| ADL disability | trajectory groups | trajectory groups, sociodemographic confounders, health behaviors, comorbidities | trajectory groups,  sociodemographic confounders, health behaviors, comorbidities,  baseline MMSE, GDS score | trajectory groups,  sociodemographic confounders, health behaviors, comorbidities, baseline MMSE, GDS score, plus baseline ADL score |
| IADL disability | trajectory groups | trajectory groups, sociodemographic confounders, health behaviors, comorbidities | trajectory groups,  sociodemographic confounders, health behaviors, comorbidities,  baseline MMSE, GDS score | trajectory groups,  sociodemographic confounders, health behaviors, comorbidities,  baseline MMSE, GDS score, plus baseline IADL score |
| RBD | trajectory groups | trajectory groups, sociodemographic confounders, health behaviors, comorbidities | trajectory groups,  sociodemographic confounders, health behaviors, comorbidities,  baseline MMSE, GDS, and ADL score | trajectory groups,  sociodemographic confounders, health behaviors, comorbidities,  baseline MMSE, GDS, and ADL score, plus baseline RBD score |
| Depression | trajectory groups | trajectory groups, sociodemographic confounders, health behaviors, comorbidities | trajectory groups,  sociodemographic confounders, health behaviors, comorbidities,  baseline MMSE, ADL score | trajectory groups,  sociodemographic confounders, health behaviors, comorbidities,  baseline MMSE, ADL score, plus baseline GDS score |
| MCR | trajectory groups | trajectory groups, sociodemographic confounders, health behaviors, comorbidities | trajectory groups,  sociodemographic confounders, health behaviors, comorbidities,  baseline MMSE, GDS, and ADL score | trajectory groups,  sociodemographic confounders, health behaviors, comorbidities,  baseline MMSE, GDS, and ADL score, plus baseline frailty status |

Abbreviations: ADL, activities of daily living; GDS, Geriatric Depression Scale; IADL, instrumental activities of daily living; MCR, motor cognitive risk syndrome; MMSE, Mini-Mental State Examination; RBD, rapid eye movement behavior disorders.
